# Salivary Metabolites Profiling in Primary Sjögren Syndrome: Identifying Purine as a Potential Biomarker

**DOI:** 10.21315/mjms-09-2025-629

**Published:** 2026-04-30

**Authors:** Fatin Izzatti Amalina Abd Aziz, Jasmin Raja, Anis Rageh Al-Maleki, Anand Ramanathan

**Affiliations:** 1 Department of Oral Maxillofacial and Clinical Sciences, Faculty of Dentistry, Universiti Malaya, Kuala Lumpur, Malaysia; 2 Division of Rheumatology, Faculty of Medicine, Universiti Malaya, Kuala Lumpur, Malaysia; 3 Department of Medical Microbiology, Faculty of Medicine, Universiti Malaya, Kuala Lumpur, Malaysia; 4 Oral Cancer Research and Coordinating Centre, Faculty of Dentistry, Universiti Malaya, Kuala Lumpur, Malaysia; 5 MAHSA University, Jenjarom, Selangor, Malaysia

**Keywords:** primary Sjögren syndrome, salivary biomarkers, metabolites, liquid chromatography-mass spectrometry, purine

## Abstract

**Background:**

Salivary biomarkers such as proteins, metabolites, hormones, and nucleic acids can provide biological information for a variety of medical problems, such as cancer, stress, systemic disorders, and neurodegenerative and infectious diseases. The proteome and metabolomic alterations observed in saliva appear to match those observed in blood, reflecting the cells’ cellular activity and physiological status.

**Methods:**

This is a case-control study that included 10 individuals [5 primary Sjögren syndrome (pSS) and 5 healthy controls (HCs)], each contributing two salivary matrices (unstimulated saliva and oral rinse), resulting in a total of 20 samples for metabolomic analysis (*n* = 20 samples from *n* = 10 participants). Salivary metabolite profiling was performed using liquid chromatography quadrupole time-of-flight mass spectrometry (LC-Q-TOF-MS), with the HMDB, BRMB, and METLIN databases used for metabolite identification. Chemometric analysis (Unscrambler software) and statistical analysis (SPSS) were used to assess the diagnostic potential of the identified metabolites.

**Results:**

LC-Q-TOF-MS identified 269 metabolites, with purine significantly upregulated in pSS patients compared to HCs (*P* = 0.01). Upregulation of purine metabolism may indicate an inflammatory response in pSS patients.

**Conclusion:**

In this study, we found that there was a significant difference in the salivary metabolites profile (purine) between patients with pSS and HCs; thus, analysing purine metabolites in saliva may serve as a potential non-invasive candidate biomarker that warrants validation in larger cohorts for discriminating between patients with pSS from HCs and monitoring disease progression.

## Introduction

Sjögren syndrome (SS) is a chronic autoimmune disease characterised by the inflammation and mononuclear cell infiltration of exocrine glands. It primarily affects the lacrimal and salivary glands (1) and presents in two forms – primary SS (pSS), which occurs in the absence of other connective tissue diseases, and secondary SS, which is associated with other autoimmune conditions (2). The disease predominantly affects women, with a female-to-male ratio of 9:1 (3), particularly Caucasians, in their fourth or fifth decade of life (4). SS also leads to symptoms such as dry mouth, dry eyes, pain and fatigue, significantly impairing quality of life. Early diagnosis and treatment are crucial to managing symptoms and preventing complications (5). However, there is no single definitive test for pSS diagnosis, and the 2016 American College of Rheumatology/European Alliance of Associations for Rheumatology (ACR/EULAR) classification criteria rely on a combination of serological, histopathological and functional assessments (6).

Saliva, a complex biofluid reflecting both local and systemic health (7), offers a promising non-invasive alternative for biomarker discovery in pSS. Advances in proteomics and metabolomics—particularly using mass spectrometry (MS) and nuclear magnetic resonance (NMR)—have facilitated the identification of potential salivary biomarkers. However, previous studies (8–14) have shown inconsistent results, with variations in metabolites, such as fucose, glycine, butyrate and proline, between pSS patients and healthy controls (HCs). These discrepancies highlight the need for further research to validate and standardise salivary metabolite profiles. Therefore, this study aims to profile salivary metabolites that can effectively differentiate pSS patients from healthy individuals, potentially contributing to the development of a simpler, non-invasive diagnostic approach.

## Methods

### Study Design

This investigation was conducted as a pilot study to explore the feasibility of salivary metabolite profiling in pSS using liquid chromatography quadrupole time-of-flight mass spectrometry (LC-Q-TOF-MS). This investigation was conducted as a pilot exploratory study to assess the feasibility of salivary metabolite profiling in pSS using LC-Q-TOF-MS. The sample size (*n* = 5 per group) was determined based on the availability of well-characterised pSS patients fulfilling the 2016 ACR/EULAR criteria and matched HCs during the recruitment period, as well as the exploratory nature of untargeted metabolomics, where small discovery cohorts are commonly used to generate hypotheses and identify candidate metabolites for subsequent validation in larger, adequately powered studies. The primary aim was to generate preliminary data to guide the design and power calculation of a larger, adequately powered validation study.

### Recruitment, Assessment and Saliva Collection

Subjects were recruited from the Department of Oral and Maxillofacial Clinical Science, Faculty of Dentistry, Universiti Malaya, with informed consent. Medical histories were reviewed, and pSS patients were selected based on the 2016 ACR/EULAR classification. Dental caries and periodontal status were assessed using a modified International Caries Detection and Assessment System (ICDAS) and a 6-point pocket chart (6PPC). Oral dryness was evaluated using the Challacombe scale (15).

Unstimulated saliva (US) and oral rinse (OR) samples were collected from five pSS patients and five HCs, all aged 18 years or older. The HCs had no autoimmune or mucosal diseases. The exclusion criteria included factors such as smoking, active oral infection, prior head and neck radiotherapy, certain systemic conditions, specific medication use, recent chemotherapy and recent clinical trial participation.

Participants refrained from eating, drinking, smoking and oral hygiene for 30 min before sample collection, which occurred in the morning. The US was collected via passive drooling, while OR involved rinsing the mouth with saline and spitting it out in a Falcon tube. Samples were kept on ice, transported and stored at −80°C until analysis.

### Preparation of Metabolite Samples

Saliva samples for LC-Q-TOF-MS were prepared following Rosli et al. (16). Fresh US and OR samples were centrifuged at 4,000 rpm for 1 h at 4°C, and supernatants were stored at −80°C. For analysis, 1 mL of thawed saliva was mixed with 4 mL methanol (1:4), vortexed, chilled at −20°C, and centrifuged at 10,000 rpm. The supernatant was dried using a refrigerated CentriVap before storage at −80°C.

### LC-Q-TOF Analysis of Metabolites

Metabolite profiling was conducted using an Agilent Infinity LC system coupled to a 6520 QTOF-MS equipped with a Dual Agilent Jet Stream electrospray ionisation (AJS-ESI) source (Agilent Technologies Inc., Santa Clara, CA, USA). Separation was performed on an Agilent Zorbax Eclipse Plus C18 column at 40°C to 45°C. A linear gradient of acetonitrile and water with formic acid was applied for 23 min. Each 2 μL sample was injected three times in the positive ion mode. Key ESI settings included 3.0 kV voltage, 300°C gas temperature and a flow rate of 0.5 mL/min. Sample blanks were run to prevent carryover. Instrument control and data acquisition were performed using MassHunter software (Agilent Technologies Inc., Santa Clara, CA, USA).

### Processing and Statistical Analysis of Metabolite Data

Each participant contributed one US and one OR sample. For between-group comparisons, analyses were performed separately within each matrix (US: pSS vs. HCs; OR: pSS vs. HCs), with the individual participant as the unit of analysis (*n* = 5 per group). Comparisons between US and OR within the same group were considered paired observations from the same individuals and were used descriptively and in multivariate analyses (principal component analysis [PCA]) to examine the overall similarity of metabolic profiles. We acknowledge that US and OR samples from the same individual are not statistically independent and have interpreted these within-subject comparisons cautiously.

Raw data were processed using MassHunter Qualitative Analysis for molecular feature extraction, with a ±5 ppm mass accuracy. Features were aligned and analysed in Mass Profiler Professional (MPP) using the normalisation to median abundance technique. PCA was used to assess group separation. Differential metabolites were identified using a one-way ANOVA with Benjamini–Hochberg False Discovery Rate (FDR) (1%), fold change ≥ 2 and *P* < 0.05, supplemented by *t*-tests. Metabolite identification was conducted using MPP’s ID browser against the METLIN Accurate Mass-Personal Compound Database and Library (AM-PCDL ) database.

## Results

This study included participants aged 18 to 62 years, with a mean age of 44.8 years. Each pSS patient was matched with HCs based on gender, age and race. All five pSS patients tested positive for the anti-SSA (Ro) antibody, with one showing strong positivity. Additionally, four patients had Schirmer test results of ≤5 mm/5 min, and three exhibited US flow rates below 0.1 mL/min. All pSS participants met the 2016 ACR/EULAR classification criteria, each scoring at least 4 points, thus confirming their diagnosis.

A descriptive statistical analysis was carried out for both groups in both types of samples (*n* = 20). A total of 269 metabolites were obtained when comparing all groups. Among the 269 metabolites identified, 84 compounds matched with the METLIN Accurate Mass Retention Time Personal Compound Database and Library (AMRT PCDL) database and 175 metabolites were given as chemical formulae. Figure 1 depicts the metabolite regulation among the 84 compounds that matched the METLIN AMRT PCDL database. Heat maps and clustering based on Euclidean distance were generated using MetaboAnalyst version 5, with significance defined as *P* < 0.05 and a fold-change cut-off of 2.

### US of HCs vs. OR of HCs

Unsupervised PCA was performed for sample quality control and to assess variations in metabolic profiles between groups by reducing data dimensionality. The descriptive analysis of US and OR from HCs (*n* = 5 each) identified 28 metabolites—20 upregulated and 8 downregulated (Supplementary Table 1). Six compounds matched the METLIN AMRT PCDL database, while 17 were identified using chemical formulae. The PCA plot (Figure 2) shows overlapping clusters, indicating minimal group separation.

### US of pSS vs. OR of pSS

A descriptive analysis between the US and OR of pSS patients (*n* = 5 each) identified 57 metabolites—29 upregulated and 28 downregulated (Supplementary Table 2). Of these, 12 matched the METLIN AMRT PCDL database, and 37 were identified by chemical formulae. The three dimensions PCA plot (Figure 3) comparing OR and US samples from pSS patients demonstrated substantial overlap between the two groups, indicating broadly similar metabolite profiles. Nonetheless, a few data points were positioned away from the main clusters, suggesting the presence of unique metabolites specific to one sample type.

### US of HCs vs. US of pSS

Descriptive analysis comparing the US of HCs and pSS (*n* = 5 each) identified 29 metabolites—11 upregulated and 18 downregulated (Supplementary Table 3). Nine metabolites matched the METLIN AMRT PCDL database, while 18 were identified using chemical formulae. The three dimensions PCA plot (Figure 4) comparing US samples from HCs and pSS patients revealed predominantly overlapping clusters, reflecting an overall similarity in metabolic composition. However, distinct outlier points were noted, which may represent key metabolic differences between the groups, highlighting unique features in those specific data points.

### OR of HCs vs. OR of pSS

Descriptive analysis comparing the OR of HCs and pSS (*n* = 5 each) identified 53 metabolites—28 upregulated and 25 downregulated (Supplementary Table 4). Ten metabolites matched the METLIN AMRT PCDL database, while 35 were identified using chemical formulae. The PCA plot (Figure 5) revealed overlapping clusters, suggesting overall similarity between the groups, while distinct data points indicated key metabolic differences.

In comparing the US and OR samples, most metabolites were found in both types, although some were unique to either US or OR. The top five metabolites for each group—US of HCs, US of pSS, OR of HCs and OR of pSS—are listed in Tables 1 and 2.

In the US samples, the top three metabolites found in pSS were purine, diethyl maleate and epicatechin 3-O-beta-D-glucopyranoside, while the top three in HCs were quassin, levoamine (chloramphenicol D base) and 5-L-glutamyl-taurine. Purine was also present in pSS when using OR samples, along with orthothymotinic acid and diethyl maleate.

A one-way ANOVA comparing the US of HCs vs. pSS and the OR of HCs vs. pSS revealed that only one metabolite, purine, reached statistical significance in the US matrix (*P* = 0.010), with an estimated fold change of 618.6 when calculated as the ratio of mean normalised intensity in pSS vs. HCs samples. Inspection of the underlying intensity values did not identify single extreme outliers driving this difference; however, given the very small sample size, this effect size should be interpreted with caution, as it may be unstable. In the OR samples, purine showed a similar direction of change but did not reach statistical significance (*P* = 0.310). Purine was upregulated in the pSS group compared to the HCs in the US samples (Figure 6). While purine was present in both the US and OR samples of HCs, it did not rank among the top five metabolites in these groups.

## Discussion

In this study, two types of samples, US and OR, were collected from two groups: HCs and patients with pSS. Metabolic profiling of these samples was performed using LC-Q-TOF-MS. Further analysis identified differences in metabolites between the HCS and pSS groups, and a statistical analysis was conducted to determine any significant differences in the metabolites between the two groups.

Several studies have reported variations in the salivary metabolic profiles of stimulated and US samples (17–20). Maruyama et al. (19) examined the metabolic profiles of mouth-rinsed water, stimulated saliva and US from healthy individuals using MS-based metabolomics. Of the 153 metabolites studied, some were found in only one or two saliva sample types. Figueira et al. (17) compared saliva from parotid, stimulated and unstimulated glands using LC-MS and targeted NMR spectroscopy, showing that while the same compounds were found, the metabolite composition ratios differed across saliva collection methods. Takeda et al. (20), using 1H-NMR, found that US samples had higher concentrations of almost all metabolites compared to stimulated samples in a cohort of healthy males. In addition, male samples contained higher levels of several metabolites than female samples. These findings suggest that comparing the metabolic profiles of US and stimulated saliva from pSS patients and HCs may provide complementary insights and should be explored in future studies.

This research shows that both the US and OR samples showed potential for reflecting individual differences and contained similar metabolites. PCA plot analysis comparing OR and US in HCs revealed overlapping compounds, highlighting the similarities between the two sample types. This is consistent with findings from other studies (18, 19), in which mouth rinse samples contained the same metabolites as US and stimulated saliva. However, some metabolites were undetected in the OR samples, likely due to the dilution of low-concentration metabolites in the oral cavity. Conversely, certain metabolites unique to OR may be attributed to the rinsing process. Overall, while US was found to be more suitable for analysing salivary metabolites in both pSS and healthy subjects, OR can be a useful alternative for pSS patients with severe dry mouth, where saliva collection is challenging. As OR may miss some metabolites present in US and introduce others from rinsing, its results should be interpreted with caution.

Purine upregulation was observed in the pSS group, suggesting metabolic disruption in the purine pathways relative to HCs. Disruptions in purine metabolism are associated with conditions such as gout, where hyperuricemia leads to crystal formation, potentially causing arthritis (21). Numerous immune cells, including monocytes/macrophages, T-lymphocytes, B-lymphocytes and microglial cells, express P2X7R, a ligand-gated non-selective positive ion channel (22). P2X7R is the P2R family member that is most frequently implicated in the initiation and maintenance of inflammatory pathways. It also regulates inflammatory responses by releasing cytokines, such as interleukin (IL)-1β. In pSS, purinergic receptor P2X ligand-gated ion channel 7 (P2X7R) mRNA and protein levels were significantly higher in peripheral blood mononuclear cells than in healthy individuals (23). P2X7R is involved in the development of pSS and, through the activation of the NALP3 inflammasome, triggers the release of proinflammatory cytokines IL-1β and IL-18 from monocytes/macrophages, contributing to various inflammatory conditions (24). Similar results were reported by Li et al. (14), with abnormalities in purine metabolism observed and upregulation found in pSS patients compared to healthy subjects. This upregulation of purine metabolism may indicate an inflammatory response in pSS patients through the NALP3 inflammasome and various proinflammatory cytokines.

The identification of purine as a suggested salivary biomarker for pSS leads to the possibility of developing a rapid, non-invasive diagnostic tool. In future clinical workflows, the targeted quantification of salivary purine could be performed using portable or bench-top LC-MS/MS systems or adapted into enzyme-based colourimetric or biosensor assays for point-of-care use. This approach would allow early screening in dental or primary care settings where patients frequently present with xerostomia. This would enable earlier referrals for rheumatologic evaluation. Additionally, purine levels could be monitored longitudinally to assess disease progression or treatment response and may potentially reduce reliance on invasive procedures, such as salivary gland biopsy. Validation in larger, multi-centre cohorts, along with the determination of diagnostic cut-off values, will be essential to integrate this biomarker into clinical decision-making algorithms.

Additionally, gamma-L-glutamyl-taurine (litoralon) was detected exclusively in the US of HCs. This dipeptide, containing taurine, is known for its anti-inflammatory effects, including the suppression of proinflammatory cytokines and reactive oxygen species (25), embodying a protective role in maintaining the immune balance.

In contrast, diethyl maleate, a glutathione-depleting agent, was identified in the US of pSS patients. By reducing glutathione (GSH) levels, diethyl maleate may exacerbate oxidative stress and cellular damage, further contributing to the inflammatory environment in pSS (26).

Overall, while gamma-L-glutamyltaurine and diethyl maleate offer insights into potential anti-inflammatory and oxidative stress pathways, the most notable finding is the upregulation of purine metabolism in pSS, supporting its possible role in driving inflammation through purinergic signalling and inflammasome activation.

## Conclusion

Using LC-Q-TOF-MS, this study identified purine as the only salivary metabolite significantly differing between pSS patients and HCs, suggesting its role as a non-invasive candidate biomarker for the disease. The sample size (5 pSS and 5 HCs) was small; therefore, the study was underpowered to detect modest effect sizes and support strong inferential claims. As such, the identification of purine should be viewed as preliminary and hypothetical. Larger studies are needed to validate the diagnostic value of purine and to explore additional metabolites to improve early detection and diagnosis in pSS. As a pilot study with a small cohort, these findings require confirmation in larger, independent populations.

## Supplementary Materials

**Supplementary Table 1:** List of metabolites of US of HCs vs. OR of HCs using LC-Q-TOF MS

Compound	FC	Regulation	Mass	Retention time
Purine + 17.719	2.1713939	Down	120.0436	17.719
Purine + 17.018333	10.301201	Up	120.0435	17.018333
Purine	3.2232714	Up	120.0436	17.3508
Levoamine (Chloramphenicol D base)	2.1821032	Up	212.0772	13.261
C8 H17 Cl O4	2.0863109	Up	212.0775	13.857
Diethyl maleate	107.949936	Up	172.0721	14.419666
C7 H10 N6 O2	2.185024	Up	210.0856	16.814
C6 H8 N6 O + 17.414	2.0449603	Up	180.0768	17.414
C6 H8 N6 O + 16.664	2.1004932	Up	180.0771	16.664
C6 H8 N6 O	2.1344075	Up	180.0766	17.111
C4 H8 N6 O2 + 16.367	2.1190543	Up	172.0721	16.367
C4 H8 N6 O2	2.140896	Up	172.0721	16.074
C4 H8 N6 O + 19.039	2.0397396	Down	156.0769	19.039
C4 H8 N6 O + 16.571669	2.2459161	Up	156.0773	16.571669
C4 H8 N6 O + 15.04	2.0946922	Up	156.0765	15.04
C4 H8 N6 O	2.1410146	Up	156.0769	14.596
C3 H10 N2 O3 S4	4.420594	Down	249.9577	16.823
C14 H20 N12 O4	2.209907	Up	420.1739	17.358667
C13 H24 N8 O8	4.585618	Down	420.1745	17.758667
C11 H24 N2 O10 + 16.075	4.8205276	Up	344.1432	16.075
C11 H24 N2 O10	4.2516375	Up	344.141	16.298
958.9557@16.668	2.0175593	Down	958.9557	16.668
8-Epiiridotrial glucoside	2.2824843	Up	344.1445	15.928
203.9261@17.41	2.1137774	Down	203.9261	17.41
109.0021@15.195499	4.032723	Down	109.0021	15.195499
101.9633@17.414	2.2423904	Up	101.9633	17.414
101.9631@17.116	2.1584604	Up	101.9631	17.116

**Supplementary Table 2:** List of metabolites of unstimulated saliva of pSS vs. OR of pSS using LC-Q-TOF MS

Compound	FC	Regulation	Mass	Retention time
Quassin + 17.42	1.894046	Down	388.1866	17.42
Quassin + 17.111	2.189327	Up	388.1883	17.111
Quassin + 16.827	2.562229	Up	388.188	16.827
Quassin	1.586545	Down	388.1869	16.72914
Purine	2.451533	Up	120.0438	16.518
Epicatechin 3-O-beta-D-glucopyranoside + 24.822	2.101554	Up	452.1338	24.822
Epicatechin 3-O-beta-D-glucopyranoside	2.147315	Up	452.1308	25.724
C9 H6 N2 S + 16.962	1.984112	Up	174.0249	16.962
C9 H6 N2 S + 15.784	1.991387	Down	174.0249	15.784
C9 H6 N2 S	1.984112	Up	174.0246	16.962
C8 H14 N6 O2 + 24.377499	1.168663	Up	226.1178	24.3775
C8 H14 N6 O2 + 24.083334	2.157123	Down	226.1186	24.08333
C8 H14 N6 O2 + 23.7875	4.635938	Up	226.1185	23.7875
C8 H14 N6 O2	4.441074	Down	226.1179	24.6835
C8 H12 O4 + 15.185	1.961133	Up	172.0723	15.185
C8 H12 O4 + 14.1455	4.536249	Up	172.0723	14.1455
C8 H12 O4 + 12.374	1.962873	Down	172.071	12.374
C8 H12 O4	1.173675	Up	172.072	14.8195
C8 H12 O3 + 18.746	1.903751	Down	156.0775	18.746
C8 H12 O3 + 18.001	3.926789	Down	156.0772	18.001
C8 H12 O3 + 17.259	2.045684	Up	156.077	17.259
C8 H12 O3 + 16.675	1.953669	Down	156.0772	16.675
C8 H12 O3	1.840361	Down	156.077	18.152
C6 H8 N6 O + 18.444	4.094946	Up	180.0773	18.444
C6 H8 N6 O + 18.153	1.992584	Down	180.0766	18.153
C6 H8 N6 O	3.653786	Down	180.0778	18.825
C6 H12 N4 O7 S	1.004671	Down	284.0448	16.446
C5 H12 N6 O2 S	2.275817	Up	220.0743	17.26
C4 H8 N6 O2 + 16.824	2.071113	Up	172.0722	16.824
C4 H8 N6 O2 + 13.6285	3.887082	Down	172.0716	13.6285
C4 H8 N6 O2	1.646691	Down	172.072	14.394
C4 H8 N6 O + 18.5	7.810862	Down	156.0774	18.5
C4 H8 N6 O + 16.519	2.034434	Up	156.0769	16.519
C4 H8 N6 O	1.820291	Down	156.0767	18.458
C13 H8 N4	2.070497	Up	220.0751	16.815
C11 H30 N8 O7	2.016223	Up	386.223	17.559
C11 H24 N2 O8 + 17.704	1.998906	Up	312.1543	17.704
C11 H24 N2 O8 + 17.417	1.982284	Down	312.1535	17.417
C11 H24 N2 O8 + 17.261002	4.050647	Up	312.1548	17.261
C11 H24 N2 O8 + 16.516	2.03747	Up	312.1543	16.516
C11 H24 N2 O8	52.12581	Down	312.1533	16.6414
C11 H12 N2 O5 S	2.257746	Down	284.0455	16.967
C10 H6 O8 S + 17.267	1.99544	Down	285.9791	17.267
C10 H6 O8 S	1.09882	Up	285.9795	16.894
Arg Glu Glu + 17.714	1.971715	Up	432.1959	17.714
Arg Glu Glu + 17.109	2.078908	Up	432.1976	17.109
Arg Glu Glu + 16.822	2.000228	Down	432.1961	16.822
Arg Glu Glu	1.853115	Down	432.1958	16.53
7,4’-Dihydroxyflavone	2.053278	Down	254.0567	16.827
203.9261@15.924	2.23588	Up	203.9261	15.924
201.8882@17.704	2.043918	Down	201.8882	17.704
201.8881@17.415	1.033097	Up	201.8881	17.415
124.9788@16.818	2.390343	Down	124.9788	16.818
124.9787@17.043251	5.169056	Down	124.9787	17.04325
124.9787@16.666	2.596588	Up	124.9787	16.666
101.9632@16.668999	5.589035	Up	101.9632	16.669
101.9631@16.827	1.956593	Down	101.9631	16.827

**Supplementary Table 3:** List of metabolites of unstimulated saliva of pSS vs. unstimulated saliva of HCs using LC-Q-TOF MS

Compound	FC	Regulation	Mass	Retention time
Purine + 17.6355	5.771073	Down	120.0435	17.6355
Purine + 17.360334	17.88476	Down	120.0436	17.36033
Purine + 17.113	2.446881	Down	120.0436	17.113
Purine + 16.920334	2.777878	Down	120.0435	16.92033
Purine + 16.518	2.309273	Up	120.0438	16.518
Purine	618.6047	Up	120.0435	16.51812
Epicatechin 3-O-beta-D-glucopyranoside	2.000037	Up	452.1338	24.822
Diethyl maleate+ 14.1455	4.218779	Up	172.0723	14.1455
Diethyl maleate	14.55509	Down	172.072	14.4436
C8 H12 O3	2.163148	Down	156.0772	16.96
C6 H8 N6 O + 17.414	2.160646	Down	180.0768	17.414
C6 H8 N6 O + 17.111	2.262593	Down	180.0766	17.111
C6 H8 N6 O + 16.5295	4.104774	Up	180.0768	16.5295
C6 H8 N6 O	21.38433	Down	180.0771	16.742
C4 H8 N6 O2	2.125749	Down	172.0715	15.033
C20 H24 O S	1.963174	Up	312.1548	17.263
C11 H30 N8 O7	1.924138	Up	386.223	17.559
C11 H24 N2 O8 + 17.708	3.82568	Up	312.1544	17.708
C11 H24 N2 O8 + 16.741	4.320632	Down	312.1537	16.741
C11 H24 N2 O8	10.78719	Down	312.1547	16.52371
C11 H24 N2 O10 + 16.079	2.471263	Down	344.1432	16.079
C11 H24 N2 O10 + 15.638	1.954402	Up	344.1423	15.638
C11 H24 N2 O10 + 15.1835	4.098767	Up	344.1439	15.1835
C11 H24 N2 O10	2.12966	Down	344.143	16.27367
C11 H12 N2 O5 S	2.579757	Down	284.0452	17.12
Butamben	2.220617	Down	386.222	17.85
Arg Glu Glu	2.283732	Down	432.1966	17.11233
203.9261@15.925667	1.648067	Up	203.9261	15.92567
101.9633@17.414	2.182078	Down	101.9633	17.414

**Supplementary Table 4:** List of metabolites of OR of pSS vs. OR of HCs using LC-Q-TOF MS

Compound	FC	Regulation	Mass	Retention time
Quassin + 18.003	2.03048	Down	388.1866	18.003
Quassin + 17.42	1.894046	Up	388.1866	17.42
Quassin + 17.262	2.256398	Down	388.1874	17.262
Quassin	29.40395	Down	388.1869	16.67042
NS-5	2.078142	Down	256.0892	16.671
MID1766:5-ethyl-5-(pentan-2-yl)-1-((2S,3R,4S,5S,6R)-3,4,5-trihydroxy-6-(hydroxymethyl) tetrahydro-2H-	1.942453	Down	388.184	16.965
Dehydroneotenone	1.941226	Up	336.0658	17.112
C9 H6 N2 S + 15.784	1.991387	Up	174.0249	15.784
C9 H6 N2 S	2.168734	Down	174.026	14.746
C8 H14 N6 O2 + 24.348	5.492312	Down	226.1185	24.348
C8 H14 N6 O2 + 24.085	4.409154	Up	226.1186	24.085
C8 H14 N6 O2 + 23.79	2.078539	Down	226.1181	23.79
C8 H14 N6 O2	4.441074	Up	226.1179	24.6835
C8 H12 O4	1.928331	Up	172.0722	14.75
C8 H12 O3 + 16.671001	4.137991	Down	156.0774	16.671
C8 H12 O3	9.08403	Down	156.0771	16.6724
C7 H10 N6 O2	1.837407	Up	210.0862	16.674
C6 H8 N6 O + 18.825	3.653786	Up	180.0778	18.825
C6 H8 N6 O	2.052988	Down	180.0768	18.457
C4 H8 N6 O2 + 13.6285	3.887082	Up	172.0716	13.6285
C4 H8 N6 O2	3.446383	Up	172.0706	14.3705
C4 H8 N6 O + 19.35	2.119529	Up	156.0771	19.35
C4 H8 N6 O + 19.039 :0	2.146284	Down	156.0769	19.039
C4 H8 N6 O + 19.039	2.103308	Down	156.0769	19.039
C4 H8 N6 O + 18.748	1.985335	Down	156.0769	18.748
C4 H8 N6 O + 18.5265	3.725406	Up	156.077	18.5265
C4 H8 N6 O + 18.458	1.90202	Up	156.0767	18.458
C4 H8 N6 O + 18.156 :2	1.91757	Down	156.0767	18.156
C4 H8 N6 O + 18.156 :1	1.935755	Down	156.0769	18.156
C4 H8 N6 O + 18.156	1.968051	Down	156.0767	18.156
C4 H8 N6 O + 16.677	4.362421	Down	156.077	16.677
C4 H8 N6 O + 15.925	1.928026	Up	156.077	15.925
C4 H8 N6 O + 14.9635	3.639004	Up	156.077	14.9635
C4 H8 N6 O + 14.736	1.923401	Up	156.0766	14.736
C4 H8 N6 O	1.820291	Up	156.0767	18.458
C13 H24 N8 O8 + 17.855999	4.763963	Down	420.1745	17.856
C13 H24 N8 O8 + 17.706	2.206255	Down	420.1725	17.706
C13 H24 N8 O8 + 17.41425	4.138999	Up	420.1722	17.41425
C13 H24 N8 O8	1.788884	Up	420.1731	17.114
C12 H18 O3 S	2.154765	Up	242.0979	13.255
C10 H6 O8 S + 17.267	1.99544	Up	285.9791	17.267
C10 H6 O8 S	2.161974	Up	285.9797	16.964
Asp Gln Met	2.031748	Up	392.1373	17.557
958.9557@16.668	2.121288	Down	958.9557	16.668
7,4’-Dihydroxyflavone	2.053278	Up	254.0567	16.827
201.8882@17.704	2.043918	Up	201.8882	17.704
2-[3-Carboxy-3-(methylammonio) propyl]-L-histidine	2.077893	Down	270.133	15.778
124.9789@17.119	4.833554	Up	124.9789	17.119
124.9788@16.819334	43.58536	Down	124.9788	16.81933
124.9787@17.0415	5.550082	Up	124.9787	17.0415
109.0027@15.035	1.975855	Down	109.0027	15.035
109.0023@15.197	1.993363	Down	109.0023	15.197
101.9631@16.827	1.956593	Up	101.9631	16.827

## Figures and Tables

**Figure 1:**
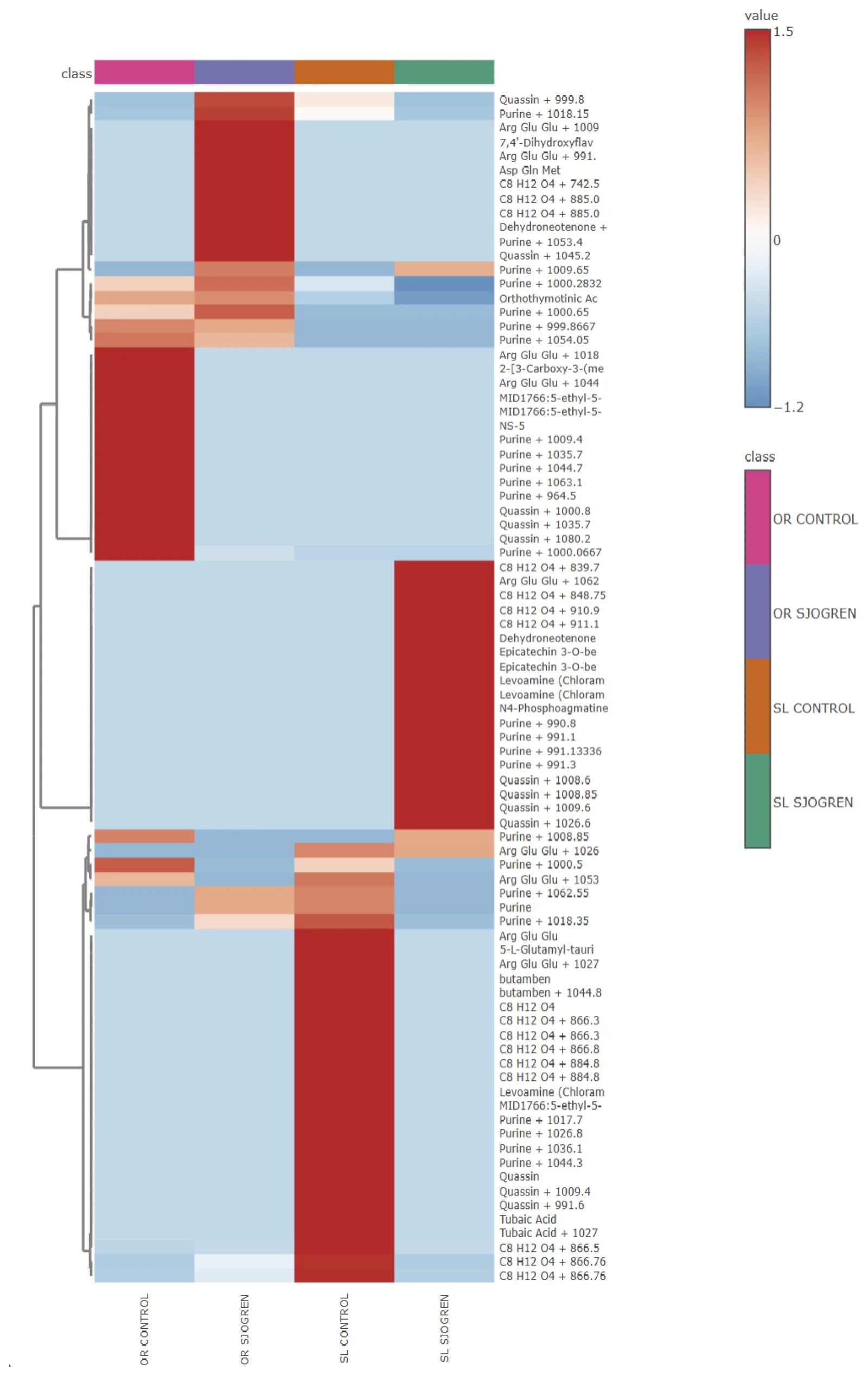
Heat map and clustering showing 84 metabolites that match with METLIN AMRT PCDL

**Figure 2:**
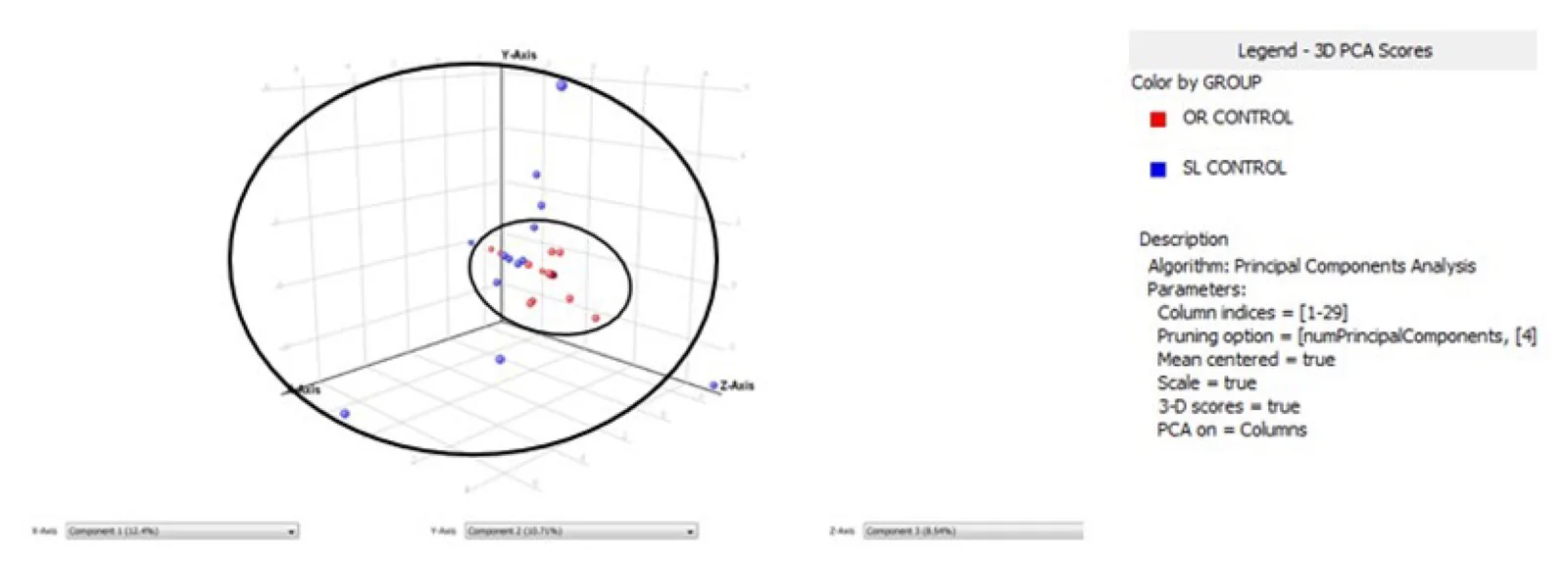
PCA in three dimensions of US of HCs (blue) vs. OR of HCs (red), created using MPP (*n* = 10)

**Figure 3:**
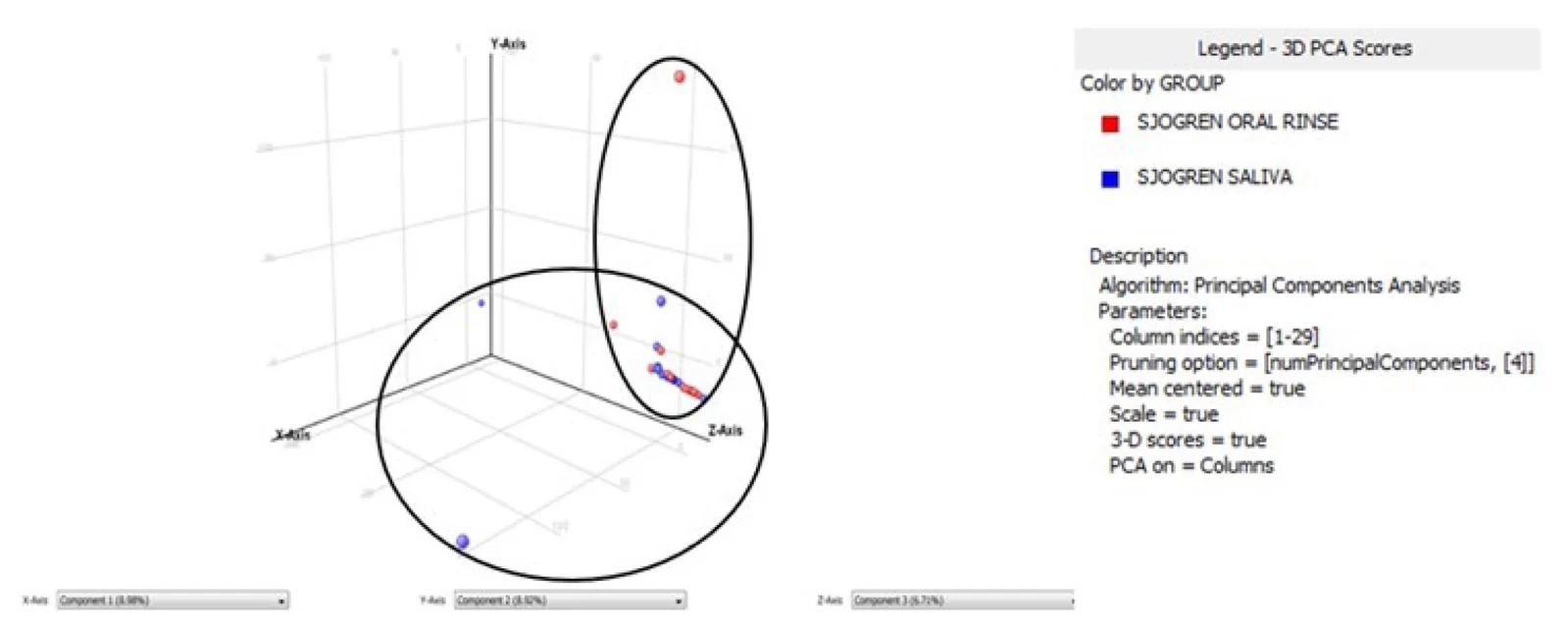
PCA in three dimensions of US of pSS (blue) vs. OR of pSS (red) created using MPP (*n* = 10)

**Figure 4:**
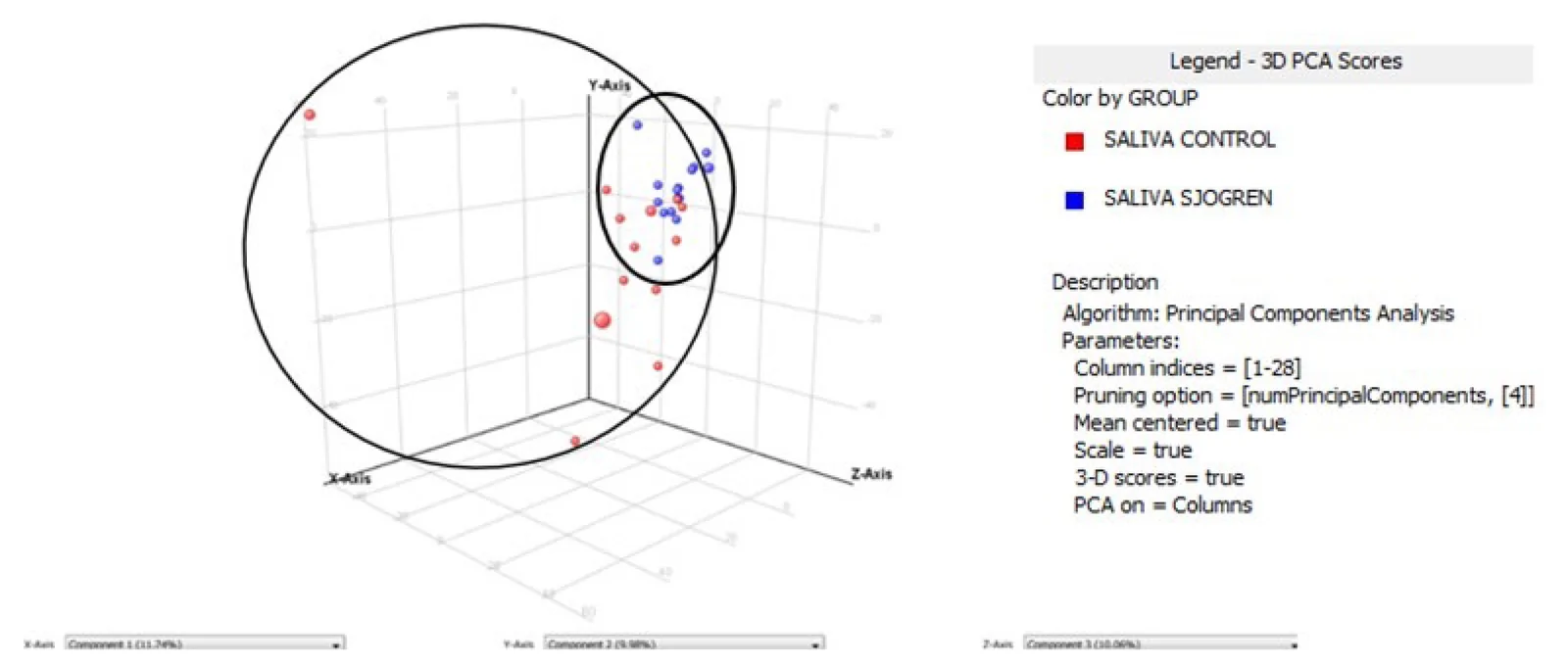
PCA in three dimensions of US of HCs (red) vs. US of pSS (blue), created using MPP (*n* = 10)

**Figure 5:**
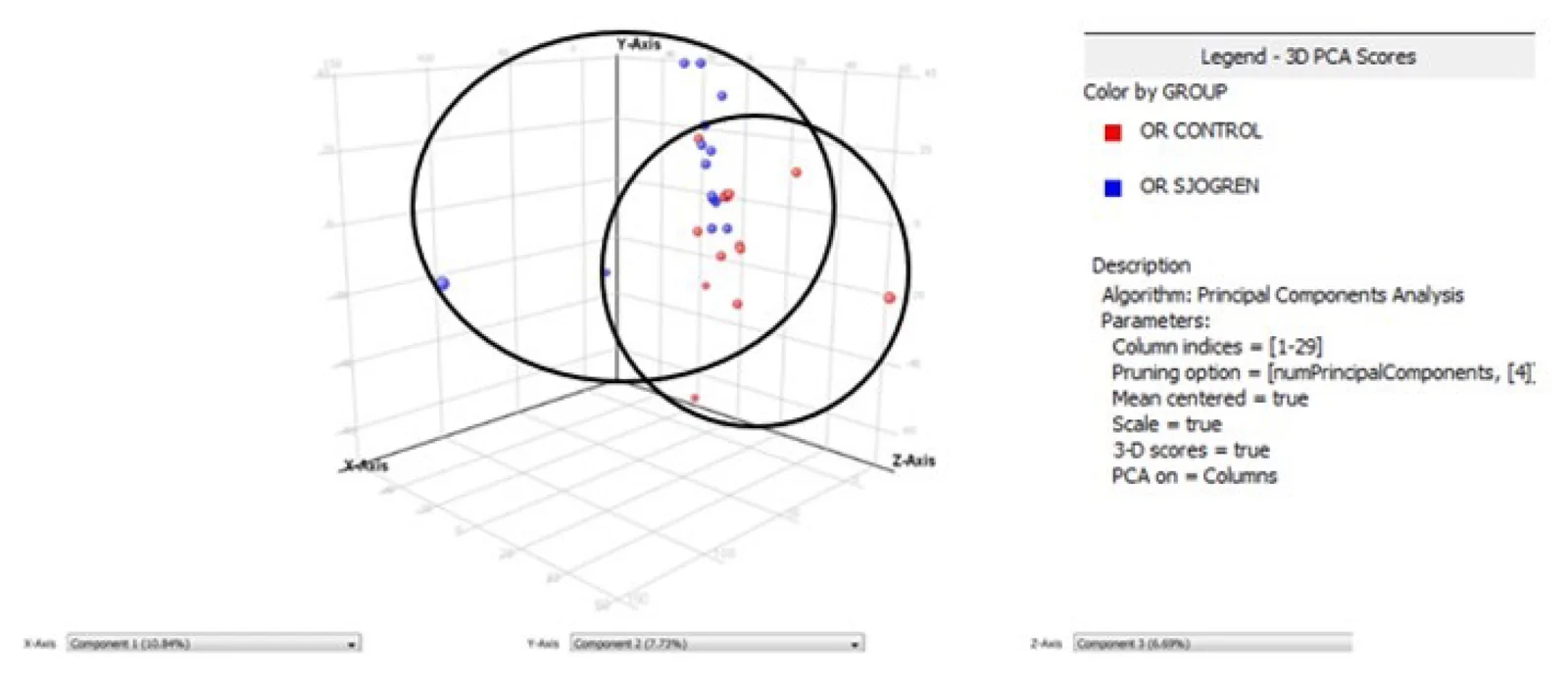
PCA in three dimensions of OR of HCs (red) vs. OR of pSS (blue), created using MPP (*n* = 10)

**Figure 6:**
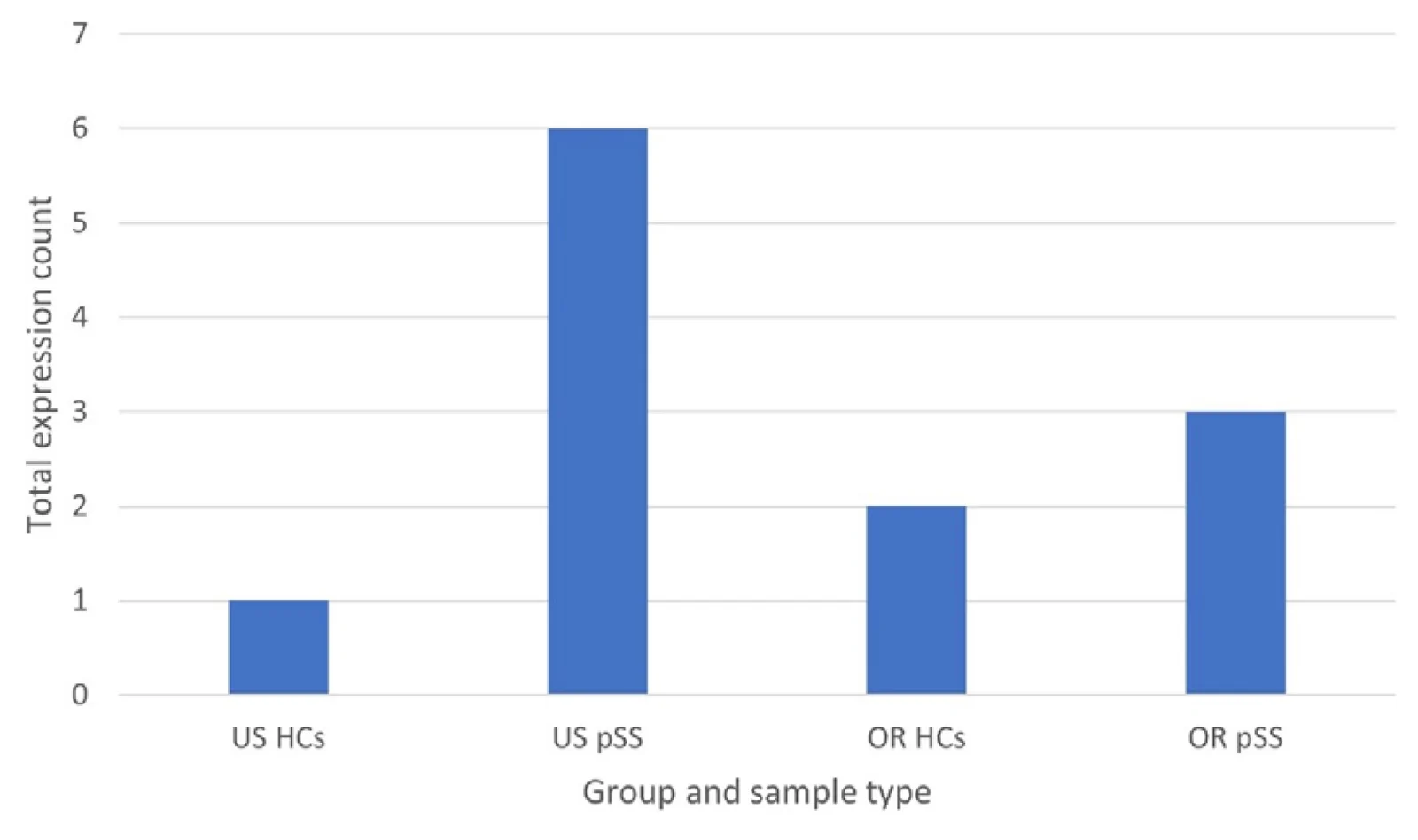
Levels of purine in both groups and both sample types, purine is present in all groups but found to be upregulated in US of pSS with *P*-value of 0.01

**Table 1:** List of the top five metabolites for both groups using US samples

No.	HCs	*P*-value	FC	Reg	pSS	*P*-value	FC	Reg
1	Quassin	0.400	2.13	Up	Purine	0.010	618.60	Up
2	Levoamine (Chloramphenicol D base)	0.309	2.18	Up	Diethyl maleate	0.185	4.21	Up
3	5-L-Glutamyl-taurine	0.400	1.94	Up	Epicatechin 3-O-beta-D-glucopyranoside	0.362	2.00	Up
4	Tubaic acid	0.400	1.99	Up	Arg Glu Glu	0.479	−2.28	Down
5	MID1766:5-ethyl-5-(pentan-2-yl)-1-((2S,3R,4S,5S,6R)-3,4,5-trihydroxy-6-(hydroxymethyl) tetrahydro-2H-	0.309	1.91	Up	Butamben	0.291	−2.20	Down

HCs= healthy controls; FC = fold change; pSS = primary Sjögren syndrome; Reg: regulation

**Table 2:** List of the top five metabolites for both groups using OR samples

No.	HCs	*P*-value	FC	Reg	pSS	*P*-value	FC	Reg
1	Quassin	0.097	−29.40	Down	Purine	0.310	2.19	Up
2	N4-Phosphoagmatine	0.400	2.18	Up	Orthothymotinic acid	0.169	1.77	Up
3	2-[3-Carboxy-3-(methylammonio) propyl]-L-histidine	0.309	1.97	Up	Quassin	0.656	2.15	Up
4	NS-5	0.259	1.97	Up	Dehydroneotenone	0.343	1.94	Up
5	MID1766:5-ethyl-5-(pentan-2-yl)-1-((2S,3R,4S,5S,6R)-3,4,5-trihydroxy-6-(hydroxymethyl) tetrahydro-2H-	0.400	1.85	Up	7,4’-Dihydroxyflavone	0.343	2.05	Up

HCs= healthy controls; FC = fold change; pSS = primary Sjögren syndrome; Reg: regulation
